# Supplementary material for: Uniform processing and analysis of IGVF massively parallel reporter assay data with MPRAsnakeflow
Source: Genome Res. 2026 Sep;36(9):1940–51. doi: 10.1101/gr.281462.125 (PMC13534247; doi:10.1101/gr.281462.125)
Supplement: Supplementary file 1 [file Supplemental_Figures.pdf]

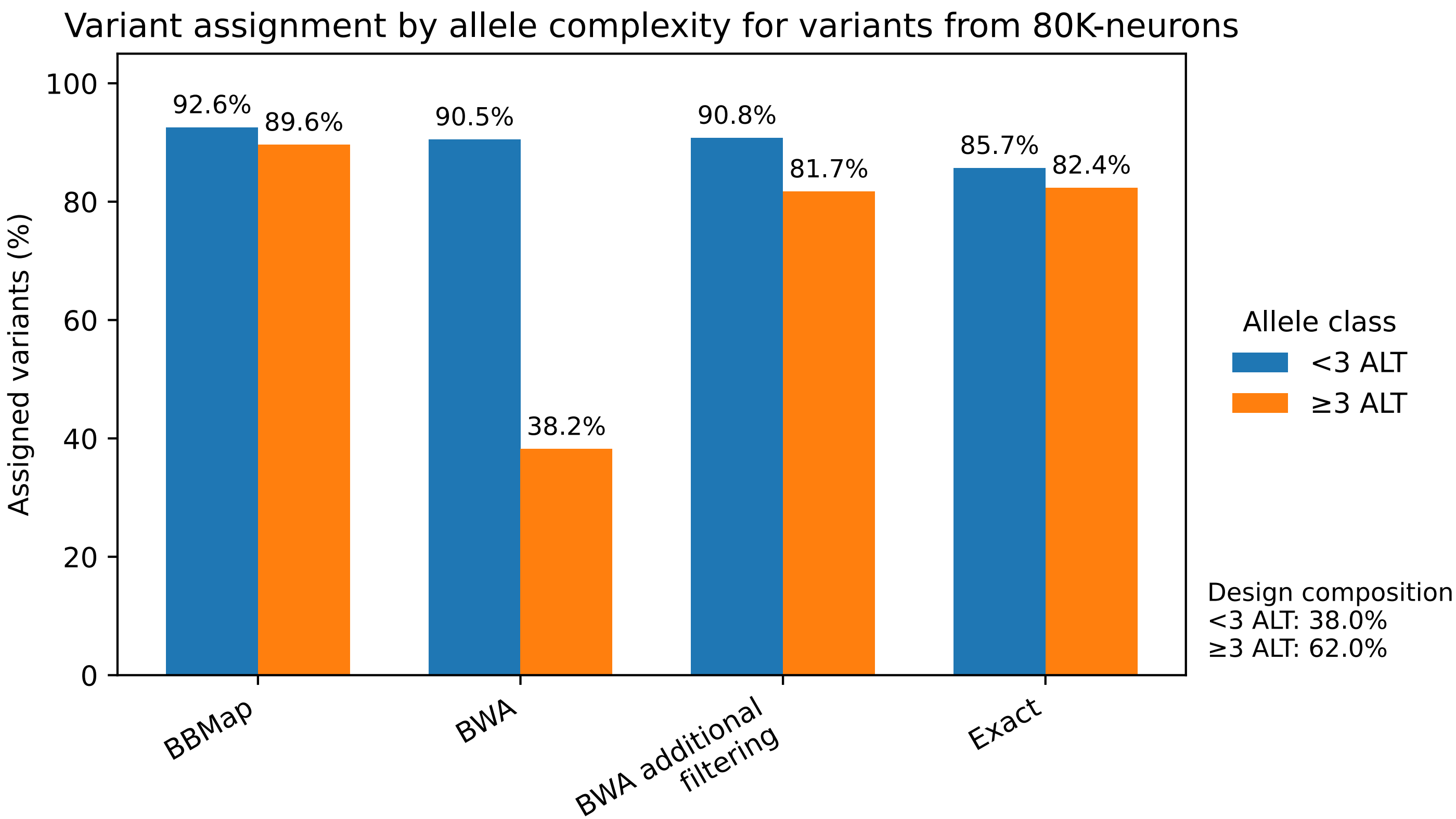

Supplemental Figure S1: Number of assigned variants for the 80K-neurons library by MPRAsnakeflow (variant-rich library). Variants are grouped by the number of ALT alleles that are designed on the same reference sequence. The left bar shows all assigned variants with reference alleles having one or two alternative alleles (ALT) designed (n=17599). Right side with three or more per reference (n=28775).

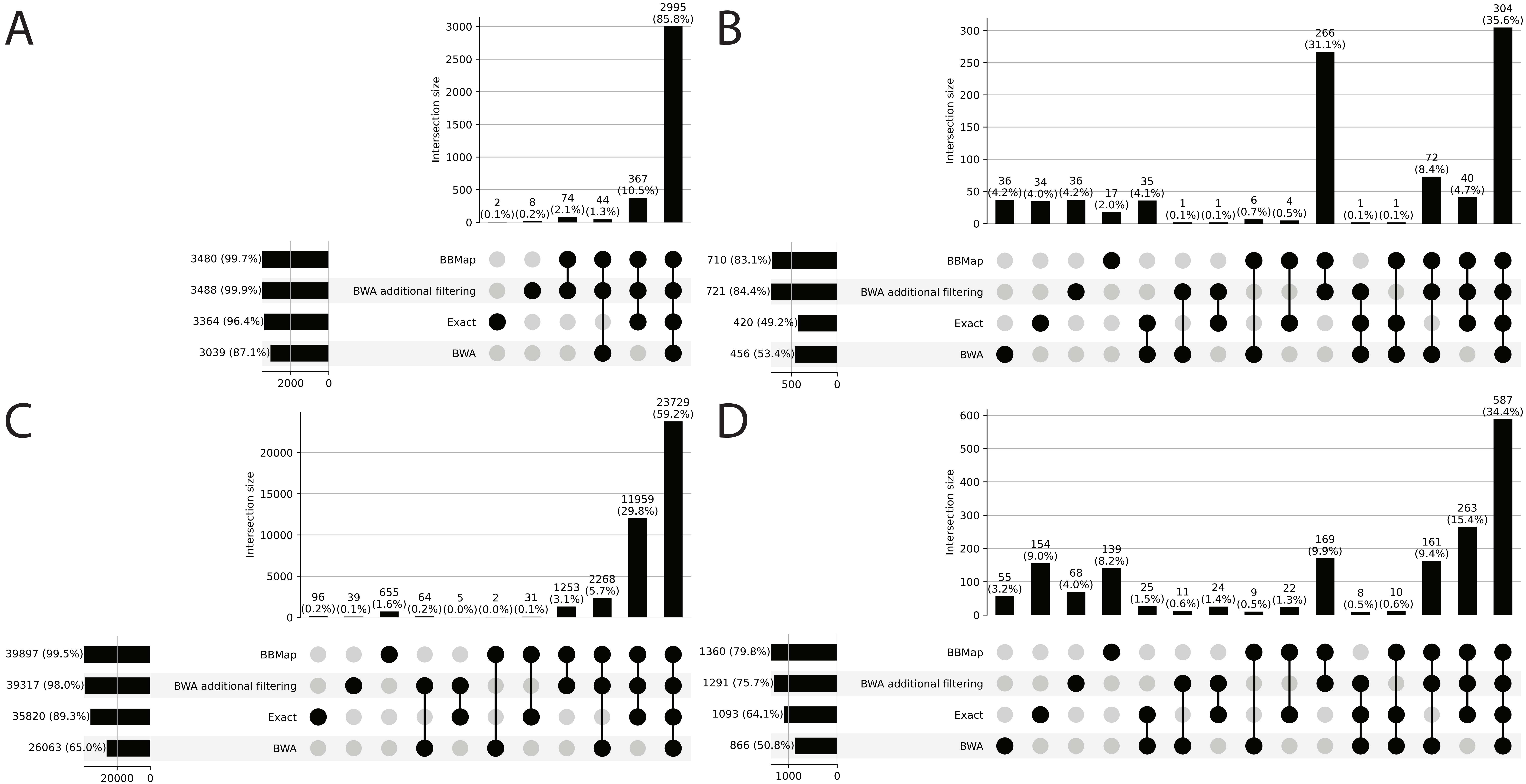

Supplemental Figure S2: Comparing BCalm results using 8K-neurons (left panels) and 80K-neurons (right panels) on the four different mapping strategies. Each of the panels contains an upset plot, which shows the set size for each mapping strategy and the size of the respective set overlap on the right. The size of the overlap is represented by the number annotated at the top of the bar. The sets used to obtain the overlap represented by the bar are shown by a black dot in the column below the bar. (A) and (C) represent the results for 8K-neurons with around 3,600 variants, and (B) and (D) show the results of 80K-neurons with around 46,000 variants. (A) and (B) show the variants with a readout between the mapping strategies, while (C) and (D) focus on the overlap between significant variants.

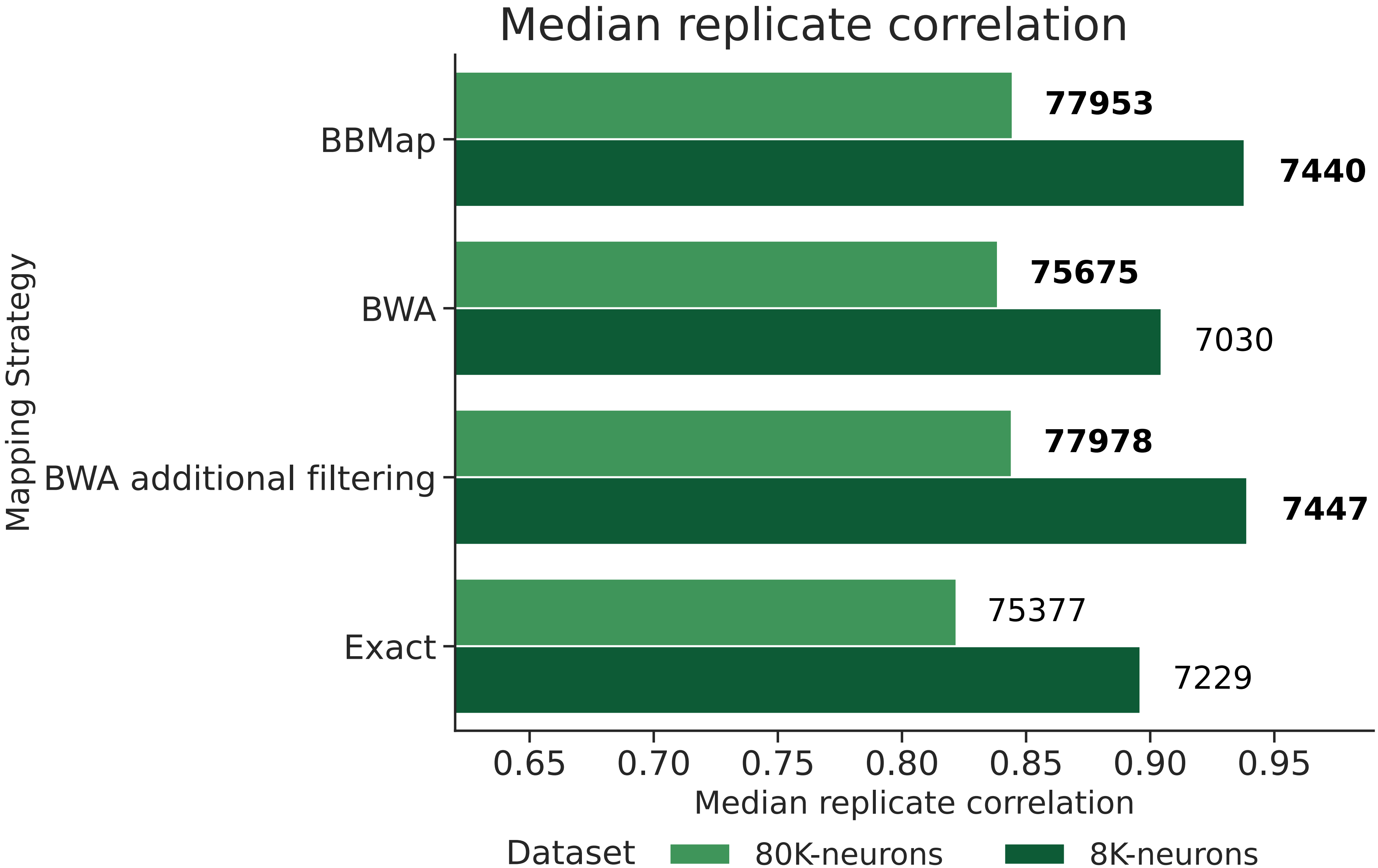

Supplemental Figure S3: Median Spearman‘s replicate (n=3) correlation of the oligo activity (log2(RNA/DNA)) for the 8K-neurons (green) and 80K-neurons (orange) dataset. The bar with the bold number of oligos included represents the mapping strategy with the highest correlation (rounded to the second digit).

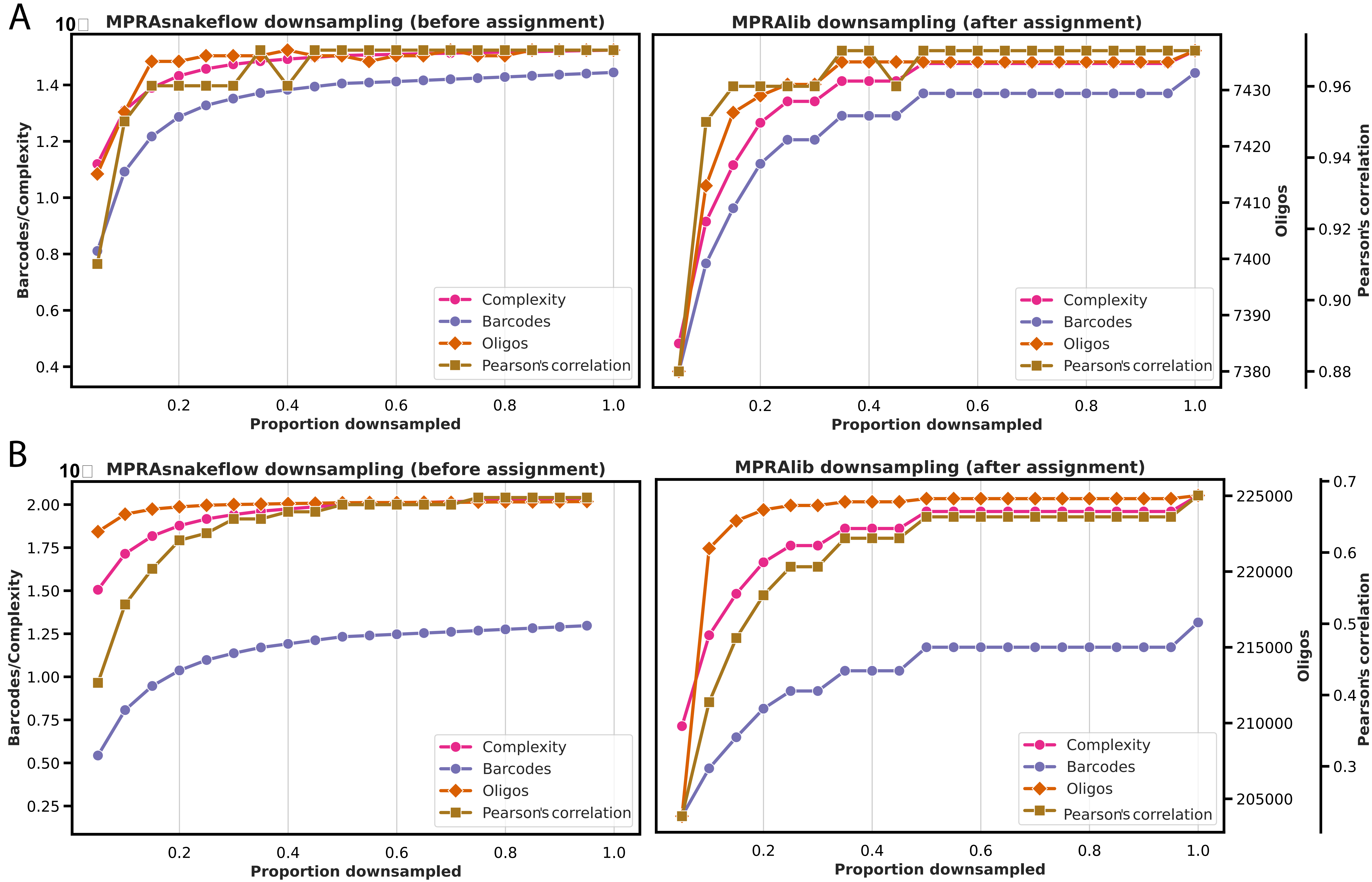

Supplemental Figure S4: Downsampling analysis for (A) 8K-neurons and (B) 240K-HepG. RNA is downsampled in equal proportions to the overall number of counts. Complexity (Lincoln index), number of barcodes as well as retained oligos and Pearson's correlation of oligo activity across replicates (median values across replicates or replicate comparisons). The left side uses RNA count downsampling in MPRAsnakeflow, which happens before the assignment (raw counts). The right side shows RNA downsampling using the MPRAlib.

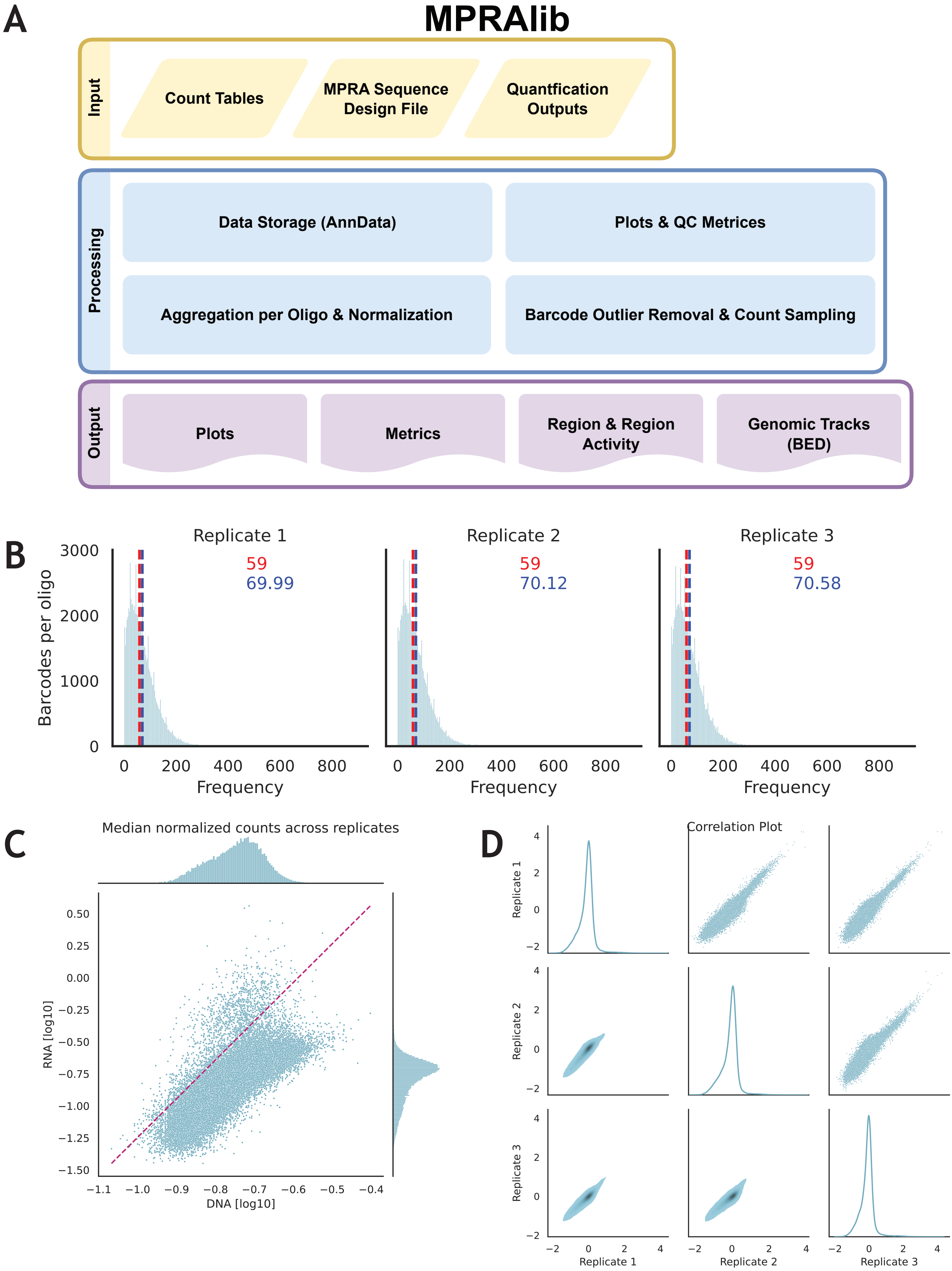

Supplemental Figure S5: (A) Overview of the python library MPRAlib. Inputs are shown in yellow, main processing steps in blue, and outputs in purple. (B) to (D) Examples of implemented plots within the python library using the 80K-neurons dataset (IGVF ID: IGVFFI0128CWLH). (B) Shows a histogram of number of barcodes per oligo per replicate. Red line/value shows the median, blue line/value the mean number of barcodes per oligo of the corresponding replicate. (C) Comparing normalized DNA and RNA counts per oligo (log10, mean normalized counts across all three replicates). This plot helps to identify if the assay is able to identify differential activity, resulting in oligos where normalized DNA counts are not equal to normalized RNA counts. (D) Correlation across replicates of log2 normalized DNA/RNA ratio (log2 activity). Right-upper part comparison with dot plots. Left-lower part density of dots. Diagonal shows the density of the activity per replicate.

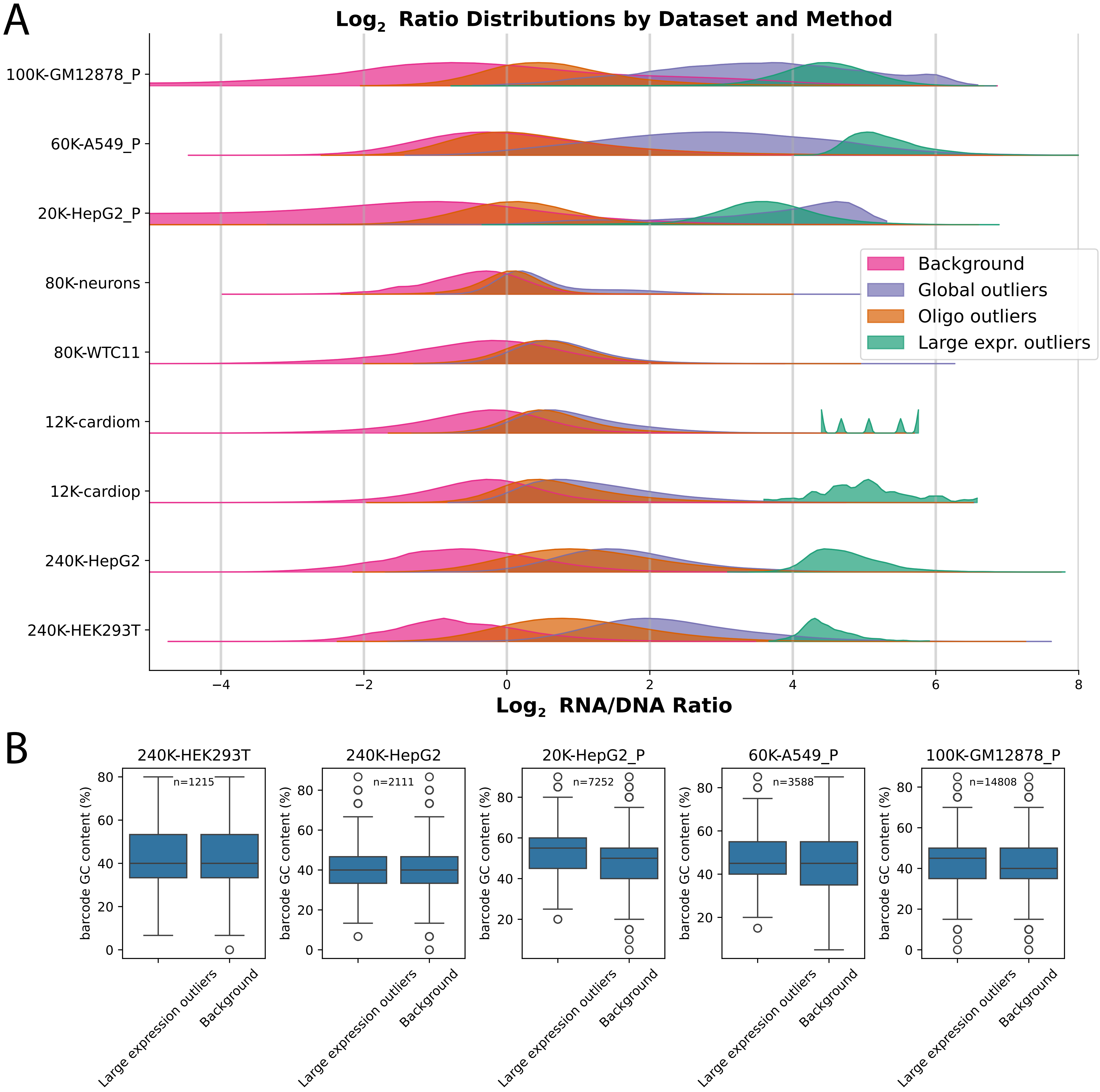

Supplemental Figure S6: (A) Distribution of log2 ratios for the eight datasets used for outlier detection grouped as background (all barcodes not flagged by any outlier method, yellow) and different outlier detection methods (global z-score, oligo-specific, and large expression in pink, green, and blue, respectively). (B) Barcode GC content comparison between large expression outliers and the background (all detected barcodes within the library). GC content is significantly different for episomal assays according to the Mann-Whitney U test (20K-HepG2_P p-value=1.12x10^{-239}, 60K-A546_P p-value=1.06x10^{-19}, and 100K-GM12878 p-value=3.48x$10^{-□1}$).

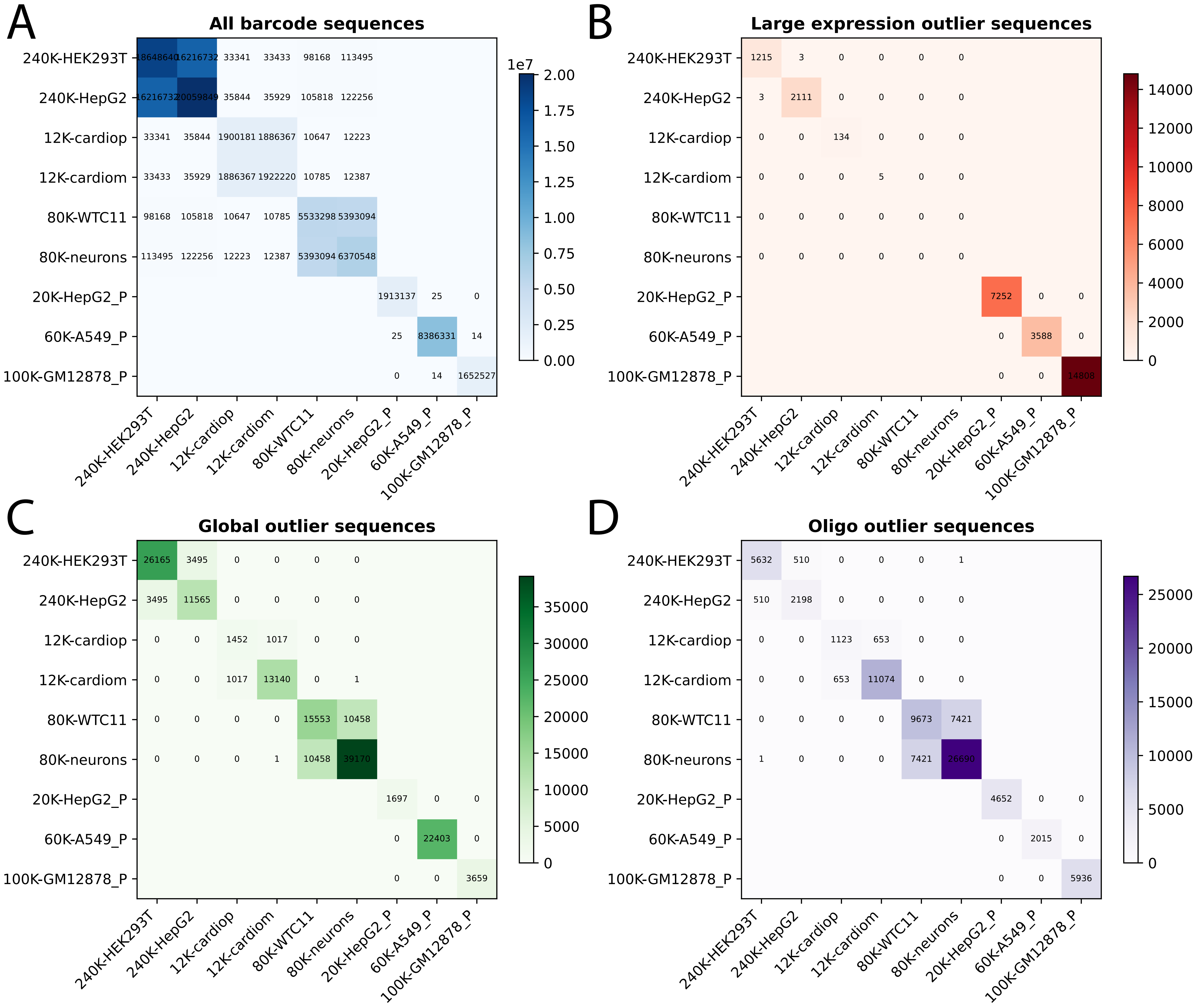

Supplemental Figure S7: Intersection matrix for (A) total barcodes detected and outlier barcodes (B - large expression, C - global outliers, D - oligo outliers) across datasets (lenti based libraries have 15 nt barcodes and episomal libraries have 20 nt barcodes).
