# Supplementary material for: Uniform processing and analysis of IGVF massively parallel reporter assay data with MPRAsnakeflow
Source: Genome Res. 2026 Sep;36(9):1940–51. doi: 10.1101/gr.281462.125 (PMC13534247; doi:10.1101/gr.281462.125)
Supplement: Supplementary file 2 [file Supplemental_Material.pdf]

# Supplemental Material

## Supplemental Note S1

Here we specify the standardized file formats around experiments. We group them into four categories. 1. Design, 2. Counts 3. Effects and 4. Genomic Effects.

### Design

#### Reporter Sequence Design

**Description:** Description of the MPRA design.
**Type:** Tab separated text with headers and without comments. Gzipped.
**Ending:** `.tsv.gz`
**Format:** custom
**Note:** * indicates required and not allowed to be empty, order of the arrays within one entry have to match (first SPDI matches first variant_pos,...), empty arrays are denoted as NA ([] or [NA] or empty string are not allowed).
**Specification:**

```
table reporter_sequence_design
"MPRA sequence design metadata format"
(
string    name*;          “A unique-within-file identifier. Starts with alphabetical
                          characters.”
string    sequence*;      “DNA string of the designed sequence. Allowed chars:
                          [A,T,G,C]”
enum      category*;      “Category of designed sequence. Allowed enums: [variant,
                          element, synthetic, scrambled]”
enum      class*;         “Class of designed sequence. Allowed enums: [test, variant
                          positive control, variant negative control, element active
                          control, element inactive control]”
string    source;         “Free form (e.g. ‘Cardio FG 2022’)."
string    ref;            “Reference genome or sequence (e.g. ‘GRCh38’).”
string    chr;            “Chromosome or contig.”
unit      start;          “0-based position of the left-most position of sequence wrt
                          the reference.”
uint      end;            “1-based position of the right-most position of sequence
                          wrt the reference.”
enum      strand;         “Strand of sequence in reference. Allowed enums: [+,-]
enum[]    variant_class;”What type of variant(s), if applicable. Allowed enums: [SNV,
                          indel]”
Uint[]    variant_pos     “0-based position of the variant(s) starts in the sequence.
                          For indels use normalized representation from SPDI.
                          Acceptable values range from 0 to length(sequence)-1.”
string[]  SPDI            “0-based, validated SPDI representation of the variant(s).”
```

```
enum[]    allele          “The coding with respect to the reference. Allowed enums:
                          [ref, alt]”
string    info            “Any additional comments.”
)
```

**Example:**

```
ID1
  ACTTAGGTTCAATTAAAGTTTTAGGTAAAGCTCACTGTTGTTGTCATTGTAATAAATGATCTAGTTTCCCGATGAATCCATATA
  ACTGGGTAATGCTATGGTCTTAACACAACCTTTATGTTATGTCAATTTCATTTAGTAAATGAAATTTCTTTGGGAGTAGGTGCT
  AACATTCTCATGTGAGTTGTATGTTTGCTGCAGTAAAAGATTATTGAGCATTAACATCTTCTAAGCAGCATGTATATGGGAAGT
  AATTAATTATACTTTCTT scrambled     element inactive control   scrambled_example   GRCh38
  NA   NA     NA     NA      NA    NA     NA     NA     NA
ID2
  GGATGATTCATGTCCCACGTGGGACAGAACAGGATGGTACGATATTTTATTATGCAACTCAGAATGACATGCAATTATTGAAAA
  CTTGTGTATTTATTTCTAGAAGTTTCTGTTTAATAGTTCCGAACTGTGGTTGGCTATAACTGAAACCACAGAAAATGAAACTGC
  AGATAAGGGGGGACCAGTGTATTTTCTGCCTATGTTTAGAAGTTTCTTTTGTTTTTTTTTTGAGATGGAGTCTCGTTCTGTCTC
  CTAGGCTAGAGTGCAGTG element       test   element_example     GRCh38 chr7   152667986
  152668256   -      NA     NA     NA     NA     NA
ID3
  TAGGTGGGGAAAGAGAATGGGAGCAGAGAGAACAGCCTGTTCCACAAAGAAAGGAGAAGAACCAAGAGTTTGGGATTAAGCATA
  AAGGTCTAATTAACATTTATTAGGTGCTAAGTGCACTGCCATGTGTCAACTCCTGTAATCCTTGAAGTAATCCTGGGAGACAGG
  CATTTCATTTCTCACCTTACAGATGGGGGCACAGAGCACAGGGAGGATCTTGAGAGGGTCCCATGGTCAGCAAGTGTTGGTGAA
  AGGATATCATCCTGGCTG variant       test   variant_reference_example  GRCh38 chr6   420853
  421123      +      ["SNV", "SNV"]       [67, 225]     ["NC_000006.12:420920:G:C",
  "NC_000006.12:421078:G:A"]      ["ref", "ref"]      NA
ID4
  TAGGTGGGGAAAGAGAATGGGAGCAGAGAGAACAGCCTGTTCCACAAAGAAAGGAGAAGAACCAAGACTTTGGGATTAAGCATA
  AAGGTCTAATTAACATTTATTAGGTGCTAAGTGCACTGCCATGTGTCAACTCCTGTAATCCTTGAAGTAATCCTGGGAGACAGG
  CATTTCATTTCTCACCTTACAGATGGGGGCACAGAGCACAGGGAGGATCTTGAGAGGGTCCCATGGTCAGCAAGTGTTGGTGAA
  AGGATATCATCCTGGCTG variant       test   variant_alt_example_1      GRCh38 chr6   420853
  421123      +      ["SNV"]       [67]   ["NC_000006.12:420920:G:C"]      ["alt"]
  NA
ID5
  TAGGTGGGGAAAGAGAATGGGAGCAGAGAGAACAGCCTGTTCCACAAAGAAAGGAGAAGAACCAAGAGTTTGGGATTAAGCATA
  AAGGTCTAATTAACATTTATTAGGTGCTAAGTGCACTGCCATGTGTCAACTCCTGTAATCCTTGAAGTAATCCTGGGAGACAGG
  CATTTCATTTCTCACCTTACAGATGGGGGCACAGAGCACAGGGAGGATCTTGAGAGGATCCCATGGTCAGCAAGTGTTGGTGAA
  AGGATATCATCCTGGCTG variant       test   variant_alt_example_2      GRCh38 chr6   420853
  421123      +      ["SNV"]       [225]  ["NC_000006.12:421078:G:A"]      ["alt"]
  NA
```

**Examples on the IGVF data portal:**
https://data.igvf.org/search/?type=File&content_type=MPRA+sequence+designs

## Reporter Barcode to Element Mapping

**Description:** Creates the link between tested oligos to associated barcodes. Can be pre-designed or learned by association sequencing.
**Type:** Tab separated text without any headers or comments. Gzipped.
**Ending:** `.tsv.gz`
**Format:** custom

**Specification:**
```
table mpra_assignment
"MPRA barcode mapping format"
(
string    barcode;         "Barcode sequence. Allowed chars [A,T,G,C]"
string    oligoName;       "Name of the oligo barcode is assigned to."
)
```
**Example:**
```
GATTTAAACTCGAAT     ID1
GCTAAGCTAAGTCCC     ID1
CGCGCATCACAACTA     ID2
ATAGCGTAATGGGCA     ID3
TGCTGTTTATGTATG     ID4
AGACTATTTACGAAA     ID5
```
**Examples on the IGVF data portal:**
https://data.igvf.org/search/?type=File&content_type=barcode+to+element+mapping

# Counts

## Reporter Experiment Barcode

**Description:** This format is needed to save the complete measurement on a barcode level of an experiment.
**Type:** Tab separated text with headers and without comments. Gzipped.
**Ending:** `.tsv.gz`
**Format:** custom
**Note:** Columns `dna_count_*` and `rna_count_*` contains a placeholder * for the name of a replicate. E.g. when 3 replicates are used (and names are `1 2 3`) the file format looks like `barcode oligo_name dna_count_1 rna_count_1 dna_count_2 rna_count_2 dna_count_3 rna_count_3`.
**Specification:**
```
table reporter_experiment_barcode
"MPRA experiment barcode level output format"
(
string    barcode;         "barcode, allowed chars [A,T,G,C]"
string    oligo_name;      "name of the oligo of the design"
uint      dna_count_*      "Number of dna counts of the barcode of a replicate ‘*’, *
                           denotes the name of the replicate (e.g. 1), when no counts
                           observed the entry is empty (or zero)"
uint    rna_count_*        "Number of rna counts of the barcode of replicate ‘*’, *
                           denotes the name of the replicate (e.g. 1), when no counts
                           observed the entry is empty (or zero)"
)
```

**Example:**

```
barcode       oligo_name    dna_count_1   rna_count_1   dna_count_2   rna_count_2
  dna_count_3 rna_count_3
GATTTAAACTCGAAT     ID1                   2      6      1      4
CGCGCATCACAACTA     ID2                   1      2
ATAGCGTAATGGGCA     ID3    12     2
TGCTGTTTATGTATG     ID4                   1      10     1      13
AGACTATTTACGAAA     ID5    2      4       3      1      4      4
```

**Examples on the IGVF data portal:**
https://data.igvf.org/search/?type=File&content_type=reporter+experiment+barcode

## Reporter Experiment

**Description:** This format is needed to save the complete measurement of an experiment.
**Type:** Tab separated text with headers and without comments. Gzipped.
**Ending:** `.tsv.gz`
**Format:** custom
**Specification:**

```
table mpra_experiment
"MPRA experiment output format"
(
string    replicate;       "Name of the replicate."
string    oligo_name;      "Name of the oligo of the design."
uint      dna_counts       "Number of raw DNA counts."
uint      rna_counts       "Number of raw RNA counts."
float     dna_normalized   "Number of normalized/scaled DNA counts (CPM), 4 decimals."
float     rna_normalized   "Number of normalized/scaled RNA counts (CPM), 4 decimals."
float     log2FoldChange   "Fold change (normalized rna/dna ratio, in log2 space),  4
                           decimals."
uint      n_bc             “Number of observed barcodes for the oligo.”
)
```

**Example:**

```
replicate     oligo_name    dna_counts    rna_counts    dna_normalized
  rna_normalized     log2FoldChange      n_bc
1 ID3   12     2      8.5714 3.3333 2.5714 1
1 ID5   2      4      1.4285 6,6666 0.2142 1
2 ID1   2      6      2.8571 3.1578 1.1052 1
2 ID2   1      2      1.4285 1.0526 0.7368 1
2 ID4   1      10     1.4285 5.2631 3.6843 1
2 ID5   3      1      4.2857 5.2631 1.2280 1
3 ID1   1      4      1.6666 1.9047 1.1428 1
3 ID4   1      13     1.6666 6.1904 3.7143 1
3 ID5   4      4      6.6666 1.9047 4.0001 1
```

**Examples on the IGVF data portal:**
https://data.igvf.org/search/?type=File&content_type=reporter+experiment

# Effects

## Reporter Element

**Description:** This format stores the raw format statistical activity analysis for elements. It is dependent on a background/negative set distribution.
**Type:** Tab separated text with headers and without comments. Gzipped.
**Ending:** `.tsv.gz`
**Format:** custom
**Specification:**

```
(
string   oligo_name;       "Name of tested oligo"
float    log2FoldChange;   "Fold change (normalized output/input ratio, in log₂ space)"
float    inputCount;       “Input count (DNA), normalized (CPM), mean across
                           replicates”
float    outputCount;      “Output count (RNA), normalized (CPM), mean across
                           replicates”
float    minusLog10PValue; "-log10 of P-value"
float    minusLog10QValue; "-log10 of Q-value (FDR)"
)
```

**Example:**

```
oligo_name    log2FoldChange      inputCount    outputCount   minusLog10PValue
  minusLog10QValue
ID1    0.1623 2.2619 2.5314 0.2218 0.1804
ID2    0.7368 2.8571 3.1578 0.0969 0.0457
ID3    2.5714 8.5714 3.3333 2.0    1.7011
ID4    1.8877 1.5476 5.7266 1.3010 0.0457
ID5    -0.6116      5.4762 3.5839 0.9586 0.5086
```

**Examples on the IGVF data portal:**
https://data.igvf.org/search/?type=File&content_type=reporter+elements

## Reporter Variant

**Description:** This format stores the raw format statistical activity analysis for variants.
**Type:** Tab separated text with headers and without comments. Gzipped.
**Ending:** `.tsv.gz`
**Format:** custom
**Specification:**

```
(
string   variant_id;       "Variant ID in Canonical SPDI format"
float    log2FoldChange;   "Fold change (alt output/input ratio divided by ref
                           output/input ratio, in log₂ space)"
float    inputCountRef;    "Input count reference allele, normalized (CPM), mean
                           across replicates"
float    outputCountRef;   "Output count reference allele, normalized (CPM), mean
                           across replicates"
```

```
float    inputCountAlt;    "Input count alternative allele, normalized (CPM), mean
                           across replicates"
float    outputCountAlt;   "Output count alternative allele, normalized (CPM), mean
                           across replicates"
Float    minusLog10PValue; "-log10 of P-value"
float    minusLog10QValue; "-log10 of Q-value (FDR)"
float    postProbEffect;   "Posterior probability of a regulatory effect"
float    CI_lower_95;      "Lower bound of a 95% interval for the variant effect"
float    CI_upper_95;      "Upper bound of a 95% interval for the variant effect"
uint     variantPos;       "0-based position of the start of the variant in the tested
                           sequence  -1 if aggregation of multiple positions within
                           tested sequences."
string   refAllele;        "normalized Canonical SPDI reference variant sequence,
                           allowed chars [A,T,G,C,0]. 0 if SPDI reference allele is
                           empty"
string   altAllele;        "normalized Canonical SPDI alternative variant sequence,
                           allowed chars [A,T,G,C,0]. 0 if SPDI alternative allele is
                           empty"
)
```

**Example:**

```
variant_id    log2FoldChange      inputCountRef outputCountRef      inputCountAlt
  outputCountAlt     minusLog10PValue    minusLog10QValue    CI_lower_95   CI_upper_95
  variantPos  refAllele    altAllele
NC_000005.10:14408058:A:G  0.5630 0.1857 0.3967 0.2038 1.1554 117.3764      112.7342
  1.0000      0.5234 0.6027 164    A      G
NC_000001.11:231734632:A:G -0.3365       0.2500 0.6502 0.1525 0.1769 81.5409
  77.1997     1.0000 -0.3665       -0.3065       240    A      G
NC_000003.12:142578094:C:T 0.1916 0.1948 0.2647 0.1880 0.4488 64.6378       60.4727
  1.0000      0.1710 0.2122 35     C      T
NC_000002.12:219267000:T:C 0.3734 0.1786 0.2030 0.1951 0.6081 63.6805       59.6404
  1.0000      0.3370 0.4098 38     T      C
NC_000005.10:14259915:C:T  0.3122 0.1965 0.2036 0.1983 0.5271 56.1467       52.2034
  1.0000      0.2781 0.3463 106    C      T
```

**Examples on the IGVF data portal:**
https://data.igvf.org/search/?type=File&content_type=reporter+variants

# Genomic Effects

## Reporter Genomic Element

**Description:** Defines the activity of an element/region within a genome. Can only be used when exact chromosome start and end location within a reference genome is available. Cannot be used for shuffled or modified elements.
**Type:** Tab separated text without comments and/or headers. Bgzipped
**Ending:** `.bed.gz`
**Format:** BED6+5

**Specification:**

```
table reporter_genomic_element
"BED6+5 MPRA element level common file format"
(
string   chrom;            "Reference sequence chromosome or scaffold"
uint     chromStart;       "Start position in chromosome, 0-based inclusive"
uint     chromEnd;         "End position in chromosome, 0-based exclusive"
string   name;             "Name of tested element or region"
uint     score;            "Indicates how dark the peak will be displayed in the
                           browser (0-1000)"
enum     strand;           "+ or - for strand, . for unknown"
float    log2FoldChange;   "Fold change (normalized output/input ratio, in log2 space)"
float    inputCount;       "Input count (DNA), normalized (CPM), mean across
                           replicates"
float    outputCount;      "Output count (RNA), normalized (CPM), mean across
                           replicates"
float    minusLog10PValue; "-log10 of P-value"
float    minusLog10QValue; "-log10 of Q-value (FDR)"
)
```

**Example:**

```
chr1	2179507	2179777	EH38E2778471	103	+	0.1414	0.1986	0.3259	2.5480	1.1580
chr1	2181843	2182113	EH38E2778476	55	+	-0.0754	0.1289	0.0716	0.0420	0.0000
chr1	2182439	2182709	EH38E2778477	18	+	0.0254	0.1993	0.2196	0.0476	0.0000
chr1	2182830	2183100	EH38E2778478	40	+	0.0552	0.2392	0.2947	0.4405	0.0000
chr1	2185027	2185297	EH38E2778480	47	+	-0.0658	0.1289	0.0762	0.0468	0.0000
chr1	2188389	2188659	EH38E2778484	31	+	-0.0426	0.1960	0.1588	0.0000	0.0000
chr1	2191262	2191532	EH38E2778490	60	+	0.0832	0.2239	0.2976	0.6756	0.0000
chr1	2191971	2192241	EH38E2778492	70	+	-0.0961	0.1871	0.1100	0.4674	0.0000
```

**Examples on the IGVF data portal:**
https://data.igvf.org/search/?type=File&content_type=reporter+genomic+element+effects

## Reporter Genomic Variant

**Description:** Defines the activity of a variant within a genome. Can only be used when exact chromosome start and end location, reference and alternative sequence (Canonical SPDI normalized format) within a reference sequence is available. Cannot be used when no Canonical SPDI is available for the variant.
**Type:** Tab separated text with comments and/or headers. Bgzipped
**Ending:** `.bed.gz`
**Format:** BED6+13

**Specification:**

```
table reporter_genomic_variant
"BED6+10 MPRA variant level common file format"
(
string   chrom;            "Reference sequence chromosome or scaffold"
uint     chromStart;       "Start position of the variant in chromosome, 0-based
                           inclusive"
uint     chromEnd;         "End position of the variant in chromosome, 0-based
                           exclusive"
string   name;             "Name of tested variant"
uint     score;            "Indicates how dark the peak will be displayed in the
                           browser (0-1000)"
enum     strand;           "+ or - for strand the variant was tested in, . for
                           unknown"
float    log2FoldChange;   "Fold change (alt output/input ratio divided by ref
                           output/input ratio, in log2 space)"
float    inputCountRef;    "Input count reference allele, normalized (CPM), mean
                           across replicates"
float    outputCountRef;   "Output count reference allele, normalized (CPM), mean
                           across replicates"
float    inputCountAlt;    "Input count alternative allele, normalized (CPM), mean
                           across replicates"
float    outputCountAlt;   "Output count alternative allele, normalized (CPM), mean
                           across replicates"
float    minusLog10PValue; "-log10 of P-value"
float    minusLog10QValue; "-log10 of Q-value (FDR)"
float    postProbEffect;   "Posterior probability of a regulatory effect"
float    CI_lower_95;      "Lower bound of a 95% interval for the variant effect"
float    CI_upper_95;      "Upper bound of a 95% interval for the variant effect"
uint     variantPos;       “0-based position of the start of the variant in the tested
                           sequence, -1 if aggregated effect over multiple positions”
string   refAllele;        “normalized Canonical SPDI reference variant sequence,
                           allowed chars [A,T,G,C,0]. 0 if SPDI reference allele is
                           empty”
string   altAllele;        “normalized Canonical SPDI alternative variant sequence,
                           allowed chars [A,T,G,C,0]. 0 if SPDI alternative allele is
                           empty”
)
```

**Example:**

```
chr1	2179590	2179591	NC_000001.11:2179590:T:C	105	+	0.0593	0.1986	0.3259	0.2090	0.3902	0.8067	0.2066	0.0063	-0.0230	0.1417	83	T	C
chr1	2191443	2191444	NC_000001.11:2191443:G:A	210	+	-0.1186	0.2239	0.2976	0.1798	0.1539	1.8291	0.6189	0.0486	-0.2129	-0.0244	181	G	A
chr1	2192014	2192015	NC_000001.11:2192014:G:T	30	+	-0.0170	0.1871	0.1100	0.1479	0.0619	0.1353	0.0272	0.0046	-0.1166	0.0826	43	G	T
chr1	2192365	2192366	NC_000001.11:2192365:T:G	7	+	-0.0043	0.1519	0.0957	0.1409	0.0839	0.1327	0.0264	0.0015	-0.0295	0.0209	116	T	G
chr1	2193141	2193142	NC_000001.11:2193141:G:A	14	+	-0.0080	0.2134	0.2164	0.1914	0.1879	0.2529	0.0515	0.0014	-0.0349	0.0189	205	G	A
chr1	2197933	2197934	NC_000001.11:2197933:C:A	22	+	-0.0127	0.1347	0.0937	0.1388	0.0880	0.4727	0.1075	0.0024	-0.0386	0.0132	71	C	A
chr1	2198006	2198007	NC_000001.11:2198006:C:G	39	+	-0.0225	0.1347	0.0937	0.1696	0.1078	1.0017	0.2703	0.0053	-0.0492	0.0043	71	C	G
```

**Examples on the IGVF data portal:**
https://data.igvf.org/search/?type=File&content_type=reporter+genomic+variant+effects

# Supplemental Note S2

All MPRA experiments used in this work with short descriptions. For an overview with references to datasets see Supplemental Table S2.

## 240K-HepG2 and 240K-HEK293T

Very large library testing variants/regions in open chromatin (screen regions) in front of genes (range between 500 and 20K nucleotides before TSS). Open chromatin regions of HepG2, HEK293T MCF-7 and stem cells are used. Variants were selected from an in-silico mutagenesis of all regions, predicted by a DNN trained on open chromatin in multiple cell-lines. Proportions of variants to be predicted to have a high, low, or neutral activity are selected.

## 100K-GM12878_P

An episomal library tested in GM12878 cells to functionally evaluate genetic variants in high, local LD for independent *cis*-expression quantitative trait loci (Abell et al. 2022). The MPRa data was released via NCBI Gene Expression Omnibus (GEO).

## 80K-WTC11 and 80K-neurons

Variants/Region design of open chromatin regions that are close to disease-causing genes related to neuronal and cardiac phenotypes. Also for all actionable genes (CAVA). All common variants (>5% AF) as well as a subset, prioritized by Enformer, of rare and singleton variants.

## 60K-A549_P

An episomal library tested in A549 cells, which was part of a MPRA set used to engineer and validate synthetic CREs capable of driving gene expression with programmed cell-type specificity (Gosai et al. 2024). This library was released via ENCODE.

## 20K-HepG2_P

An episomal library tested in HepG2 cells. Lead Genome-Wide Association Studies variants for lipid-related traits (HDL-cholesterol, LDL-cholesterol, triglycerides, and the liver enzymes ALT, AST, and GGT) were selected from large meta-analyses (Vujkovic et al. 2022; Sakaue et al. 2021; Pazoki et al. 2021; Gao et al. 2021; Kanoni et al. 2022; Graham et al. 2021). Complete signals were built by identifying variants in high linkage disequilibrium (LD; $r^2 \geq 0.8$ in European reference panel).

## 12K-cardiop and 12K-cardiom

Small library with mostly regions tested in cardiomyocytes and cardioprogenitor cells derived from WTC11.

## 8K-neurons

Small library on ASD risk variants. See the original publication for more details (Koesterich et al. 2023).

# Bibliography


Abell NS, DeGorter MK, Gloudemans MJ, Greenwald E, Smith KS, He Z, Montgomery SB. 2022. Multiple causal variants underlie genetic associations in humans. *Science* **375**: 1247–1254.

Gao C, Marcketta A, Backman JD, O'Dushlaine C, Staples J, Ferreira MAR, Lotta LA, Overton JD, Reid JG, Mirshahi T, et al. 2021. Genome-wide association analysis of serum alanine and aspartate aminotransferase, and the modifying effects of BMI in 388k European individuals. *Genet Epidemiol* **45**: 664–681.

Gosai SJ, Castro RI, Fuentes N, Butts JC, Mouri K, Alasoadura M, Kales S, Nguyen TTL, Noche RR, Rao AS, et al. 2024. Machine-guided design of cell-type-targeting cis-regulatory elements. *Nature* **634**: 1211–1220.

Graham SE, Clarke SL, Wu K-HH, Kanoni S, Zajac GJM, Ramdas S, Surakka I, Ntalla I, Vedantam S, Winkler TW, et al. 2021. The power of genetic diversity in genome-wide association studies of lipids. *Nature* **600**: 675–679.

Kanoni S, Graham SE, Wang Y, Surakka I, Ramdas S, Zhu X, Clarke SL, Bhatti KF, Vedantam S, Winkler TW, et al. 2022. Implicating genes, pleiotropy, and sexual dimorphism at blood lipid loci through multi-ancestry meta-analysis. *Genome Biol* **23**: 268.

Koesterich J, An J-Y, Inoue F, Sohota A, Ahituv N, Sanders SJ, Kreimer A. 2023. Characterization of De Novo Promoter Variants in Autism Spectrum Disorder with Massively Parallel Reporter Assays. *Int J Mol Sci* **24**: 3509.

Pazoki R, Vujkovic M, Elliott J, Evangelou E, Gill D, Ghanbari M, van der Most PJ, Pinto RC, Wielscher M, Farlik M, et al. 2021. Genetic analysis in European ancestry individuals identifies 517 loci associated with liver enzymes. *Nat Commun* **12**: 2579.

Sakaue S, Kanai M, Tanigawa Y, Karjalainen J, Kurki M, Koshiba S, Narita A, Konuma T, Yamamoto K, Akiyama M, et al. 2021. A cross-population atlas of genetic associations for 220 human phenotypes. *Nat Genet* **53**: 1415–1424.

Vujkovic M, Ramdas S, Lorenz KM, Guo X, Darlay R, Cordell HJ, He J, Gindin Y, Chung C, Myers RP, et al. 2022. A multiancestry genome-wide association study of unexplained chronic ALT elevation as a proxy for nonalcoholic fatty liver disease with histological and radiological validation. *Nat Genet* **54**: 761–771.
