# Supplementary material for: Uniform processing and analysis of IGVF massively parallel reporter assay data with MPRAsnakeflow
Source: Genome Res. 2026 Sep;36(9):1940–51. doi: 10.1101/gr.281462.125 (PMC13534247; doi:10.1101/gr.281462.125)
Supplement: Supplementary file 3 [file Supplemental_Table_S1.pdf]

Supplemental Table S1: Comparison of MPRAsnakeflow with MPRAflow.

			MPRAflow	MPRAsnakeflow
Workflow management system			Nextflow	Snakemake
Software development method			conda	conda, apptainer/singularity
Assignment workflow	Read support	Paired-end	☑	☑
		Hybrid-read (BC + Oligo)	✗	☑
		Single-end	✗	☑
		Long reads	✗	☑
	Adapter trimming		✗	☑
	Strand sensitivity		✗	☑
	Read merging		fastq-join (fixed settings)	NGmerge, fastq-join; (configurable)
	Parallelisation		☑	☑
	Mapping		BWA	BBMap, BWA, BWA + extra filtering, exact, pbmm2; (configurable)
	Assignment file		pickle format	tab separated (gz compressed)
	Summary plots		☑	☑
	Summary statistics		✗	☑
	QC report		✗	☑
Count/Experiment workflow	Read support	Paired-end	☑	☑
		Single-end	✗	☑
		UMI	☑	☑
		Without UMI	✗	☑
	Adapter trimming		✗	☑
	Custom assignment		☑ by generating special pickle file	☑
	Outlier removal		✗	☑
	Count sampling		✗	☑
	Summary plots		☑	☑
	Summary statistics		✗	☑
	QC report		✗	☑
