# Supplementary material for: Uniform processing and analysis of IGVF massively parallel reporter assay data with MPRAsnakeflow
Source: Genome Res. 2026 Sep;36(9):1940–51. doi: 10.1101/gr.281462.125 (PMC13534247; doi:10.1101/gr.281462.125)
Supplement: Supplementary file 4 [file Supplemental_Code_S1.zip › MPRAsnakeflow-0.7.0/docs/4_examples/GSE325256/GSE325256.assignment.qc_report.bbmap.default.html]

MPRAsnakeflow assignment QC report


## Table of contents

- Overview
- Overall quality metrics
- BC distribution over oligos
- Counts of barcodes and oligos

# MPRAsnakeflow assignment QC report

 Code

# Overview

The Assignment QC (Quality Control) Report is a comprehensive document designed to evaluate and ensure the quality of data or processes involved in a specific assignment. This report is generated for the GSE325256 assignment with settings defined in the config default. The barcode length for this experiment is 15 bases.

- **Forward:** data/assignment/SRR37895574\_1.fastq.gz
- **Reverse:** data/assignment/SRR37895574\_3.fastq.gz
- **Barcode:** data/assignment/SRR37895574\_2.fastq.gz
- **Design File:** data/assignment/GSM9640449\_VariantMPRAdesignFile.seq\_merged.fa

# Overall quality metrics

Table explanation

- **median assigned barcodes:** Median number of barcodes assigned to tested sequences in mapping as a quality control measure for the assignment step, whether there is sufficient barcode to oligo coverage.
- **fraction assigned oligos:** Fraction of assigned tested sequences in mapping to determine if the library during the assignment step was sufficiently recovered.

| median assigned barcodes | fraction assigned oligos |
| --- | --- |
| 878 | 0.69 |

# BC distribution over oligos

# Counts of barcodes and oligos

Table explanation

- **BCs** are the different observed barcodes in the sequencing data (not the overall barcode count/number of BC reads).
- Differences in row `BCs` between `Raw data` and `Filtered data` are due to barcodes that do not match the length of 15 bases defined in the default config of the config file.
- **Other BCs** are those barcodes where no oligo could be assigned due to mapping (e.g., due to MAPQ filter or due to multiple matches).
- **Ambiguous BCs** are those that map to a designed oligo but failed the minimum required barcodes per oligo of 3 and/or the minimum required fraction of 0.75 assigned to one unique oligo.

|  | Design | Raw data | Filtered data |
| --- | --- | --- | --- |
| Oligos | 32889 | 25925 | 22779 |
| BCs | 0 | 86143661 | 86143661 |
| Assigned BCs | 0 | 0 | 27729887 |
| Other BCs | 0 | 0 | 26027739 |
| Ambiguous BCs | 0 | 0 | 32386035 |


##### Source Code

```
---
title: "MPRAsnakeflow assignment QC report"
page-layout: full
format:
  html:
    code-fold: true
    code-tools: true
    embed-resources: true
    anchor-sections: true
    smooth-scroll: true
    toc: true
jupyter: python3
---


```{python}
#| tags: [parameters]
#| echo: false
```

```{python}
#| echo: false
# Imports
from IPython.display import display, Image, Markdown
import pandas as pd
import json
```


# Overview
The Assignment QC (Quality Control) Report is a comprehensive document designed to evaluate and
ensure the quality of data or processes involved in a specific assignment. This report is generated
for the `{python} assignment` assignment with settings defined in the config `{python} configs`. The barcode length for this experiment is `{python} bc_length` bases.

- **Forward:** `{python} fwd`
- **Reverse:** `{python} rev`
- **Barcode:** `{python} bc`
- **Design File:** `{python} design_file`


```{python}
#| echo: false

with open(f"{workdir}/{design_file_checked}") as f:
    seq_num_in_design = 0
    lines = f.readlines()
    for line in lines:
      if line.startswith(">"):
        seq_num_in_design += 1

```


# Overall quality metrics

<details>
    <summary>Table explanation</summary>

- **median assigned barcodes:** Median number of barcodes assigned to tested sequences in mapping as a quality control measure for the assignment step, whether there is sufficient barcode to oligo coverage.
- **fraction assigned oligos:** Fraction of assigned tested sequences in mapping to determine if the library during the assignment step was sufficiently recovered.

</details>

```{python}
#| echo: false
with open(f"{workdir}/{qc_metrics_file}", 'r') as file:
    qc_data = json.load(file)
df_qc = pd.DataFrame([qc_data])
df_qc.columns = df_qc.columns.str.replace("_", " ", regex=False)
#df_qc.columns = ['Value']
df_qc.style.hide(axis="index").format(precision=2)
```


# BC distribution over oligos
```{python}
#| echo: false
display(Image(f"{workdir}/{plot_file}"))
```


# Counts of barcodes and oligos

<details>
    <summary>Table explanation</summary>

- **BCs** are the different observed barcodes in the sequencing data (not the overall barcode count/number of BC reads).
- Differences in row `BCs` between `Raw data` and `Filtered data` are due to barcodes that do not match the length of `{python} bc_length` bases defined in the `{python} configs` config of the config file.
- **Other BCs** are those barcodes where no oligo could be assigned due to mapping (e.g., due to MAPQ filter or due to multiple matches).
- **Ambiguous BCs** are those that map to a designed oligo but failed the minimum required barcodes per oligo of `{python} min_support` and/or the minimum required fraction of `{python} fraction` assigned to one unique oligo.
    
</details>

```{python}
#| echo: false
import pandas as pd
from IPython.display import HTML, display

df_design = pd.DataFrame({' ': ['Oligos'], 'Design': int(seq_num_in_design)})
df_total = pd.read_csv(f"{workdir}/{statistic_all_file}", sep='\t').rename(columns={'Unnamed: 0': ' ', 'Counts': 'Raw data'})
df_filter = pd.read_csv(f"{workdir}/{statistic_filter_file}", sep='\t').rename(columns={'Unnamed: 0': ' ', 'Counts': 'Filtered data'})

# Concatenate the new row DataFrame with the original DataFrame
df = df_design.merge(df_total, on=' ', how="right")
df = df.merge(df_filter, on=' ', how="right").reset_index(drop=True).fillna(0).astype({"Design": int, "Raw data": int, "Filtered data": int})
df.style.hide(axis="index").format( precision=2)
```
```
