# Supplementary material for: Uniform processing and analysis of IGVF massively parallel reporter assay data with MPRAsnakeflow
Source: Genome Res. 2026 Sep;36(9):1940–51. doi: 10.1101/gr.281462.125 (PMC13534247; doi:10.1101/gr.281462.125)
Supplement: Supplementary file 4 [file Supplemental_Code_S1.zip › MPRAsnakeflow-0.7.0/docs/4_examples/GSE306816/GSE306816.qc_report.dpSTRHelaRNHWT.GSM9209853Element.default.html]

MPRAsnakeflow experiment QC report


## Table of contents

- Overall quality metrics
- DNA over RNA counts
- Oligo correlation
- Experiment statistic
- Histograms barcodes per oligo, counts per barcode
- Activity

# MPRAsnakeflow experiment QC report

 Code

TipGood Spearman correlation across replicates!

The data has a good Spearman correlations across replicates using a barcode threshold per oligo of 10! Spearman correlation of DNA is 0.98, RNA is 0.98 and DNA/RNA ratio is 0.97.

# Overall quality metrics

Table explanation

- **median rna read count:** Median of RNA read count for oligos that passed filtering to determine sufficient coverage in terms of read count. Value is the median of all replicates.
- **median barcodes passing filtering:** Median number of barcodes across tested sequences that passed filtering to determine if there was sufficient barcode to oligo coverage. Value is the median of all replicates.
- **pearson correlation:** The correlation of log2 RNA/DNA ratios across tested sequences as a measure of replicable activity signal. Value is the median of replicate comparisons using only oligos with >= 10 barcodes.
- **fraction oligos passing:** Fraction of tested sequences that passed filtering of the mappable sequences to determine if the designed library was sufficiently recovered. Value is the median of all replicates and using only oligos with >= 10 barcodes.

| median barcodes passing filtering | median rna read count | pearson correlation | fraction oligos passing |
| --- | --- | --- | --- |
| 9 | 495 | 0.99 | 0.46 |

# DNA over RNA counts

Plotting normalized counts of DNA vs RNA (median across replicates). Only oligos within all replicates are shown. We should see a variation within the RNA count data (along the y axis). If count data between RNA and DNA is highly correlated (e.g. follows the identity line) there is no variation between designed oligos. This is an indication that RNA is inflated with DNA and the DNA digestion before creating cDNA did not work as expected.

- Threshold 10
- All

# Oligo correlation

Oligo correlation plots of DNA, RNA and DNA/RNA ratios across replicates. First tab shows plots using (in average) 24900 oligos with a minimum number of 10 barcodes. Second tab shows all 53691 oligos that have assigned barcodes.

- Threshold 10
- All

| Condition | A | B | #Oligos A | #Oligos B | #Oligos Joined | DNA spearman | RNA spearman | Ratio spearman | DNA log2 pearson | RNA log2 pearson | Ratio log2 pearson |
| --- | --- | --- | --- | --- | --- | --- | --- | --- | --- | --- | --- |
| dpSTRHelaRNHWT | 1 | 2 | 25412 | 25369 | 24851 | 0.98 | 0.98 | 0.97 | 0.98 | 0.99 | 0.99 |
| dpSTRHelaRNHWT | 1 | 3 | 25412 | 25486 | 24938 | 0.98 | 0.98 | 0.97 | 0.98 | 0.99 | 0.99 |
| dpSTRHelaRNHWT | 2 | 3 | 25369 | 25486 | 24912 | 0.98 | 0.98 | 0.97 | 0.98 | 0.99 | 0.99 |

| Condition | A | B | #Oligos A | #Oligos B | #Oligos Joined | DNA spearman | RNA spearman | Ratio spearman | DNA log2 pearson | RNA log2 pearson | Ratio log2 pearson |
| --- | --- | --- | --- | --- | --- | --- | --- | --- | --- | --- | --- |
| dpSTRHelaRNHWT | 1 | 2 | 53770 | 53759 | 53673 | 0.97 | 0.97 | 0.92 | 0.97 | 0.98 | 0.96 |
| dpSTRHelaRNHWT | 1 | 3 | 53770 | 53794 | 53707 | 0.97 | 0.98 | 0.93 | 0.97 | 0.98 | 0.96 |
| dpSTRHelaRNHWT | 2 | 3 | 53759 | 53794 | 53693 | 0.97 | 0.98 | 0.92 | 0.97 | 0.98 | 0.96 |

- Threshold 10
- All

- DNA
- RNA
- Ratio

- DNA
- RNA
- Ratio

# Experiment statistic

The total number of oligos in this experiment is 55801 (defined by the assignment) with 1002989 associated barcodes.

In average across replicates we see 53774 from 566650 average barcodes in the count data and around 310254 barcodes where not in the assignment.

- DNA/RNA merged
- DNA/RNA individual

| condition | replicate | oligos dna/rna | matched barcodes | unknown barcodes dna/rna | % matched barcodes | total dna counts | total rna counts | avg dna counts per bc | avg rna counts per bc | barcode outlier removed | avg dna/rna barcodes per oligo |
| --- | --- | --- | --- | --- | --- | --- | --- | --- | --- | --- | --- |
| dpSTRHelaRNHWT | 1 | 53770 | 566177 | 308349 | 64.74 | 93099148 | 81412011 | 106.46 | 93.09 | 0 | 10.53 |
| dpSTRHelaRNHWT | 2 | 53759 | 565522 | 307289 | 64.79 | 95678257 | 77722471 | 109.62 | 89.05 | 0 | 10.52 |
| dpSTRHelaRNHWT | 3 | 53794 | 568251 | 315124 | 64.33 | 90081681 | 96615958 | 101.97 | 109.37 | 0 | 10.56 |

| Experiment | Barcodes | Counts | Average counts | Assigned barcodes | Assigned counts | Average assigned counts | Fraction assigned barcodes | Fraction assigned counts |
| --- | --- | --- | --- | --- | --- | --- | --- | --- |
| dpSTRHelaRNHWT.1.DNA | 1752650 | 94321785 | 53.82 | 591342 | 66570416 | 112.58 | 0.34 | 0.71 |
| dpSTRHelaRNHWT.2.DNA | 1813506 | 96993189 | 53.48 | 591571 | 68414547 | 115.65 | 0.33 | 0.71 |
| dpSTRHelaRNHWT.3.DNA | 1699844 | 91193786 | 53.65 | 590256 | 64358639 | 109.04 | 0.35 | 0.71 |
| dpSTRHelaRNHWT.1.RNA | 1883768 | 83025819 | 44.07 | 571968 | 58849143 | 102.89 | 0.30 | 0.71 |
| dpSTRHelaRNHWT.2.RNA | 1814843 | 79225854 | 43.65 | 571195 | 56178409 | 98.35 | 0.31 | 0.71 |
| dpSTRHelaRNHWT.3.RNA | 2115357 | 98633947 | 46.63 | 575048 | 69833093 | 121.44 | 0.27 | 0.71 |

# Histograms barcodes per oligo, counts per barcode

Histogramm of number of barcodes per oligo and the number of counts per barcode devidied by DNA and RNA. Median is red, mean is blue.

- Number of barcodes per oligo
- Number of counts per DNA barcode
- Number of counts per RNA barcode

# Activity

Violin and box plots of the log2 fold change of all oligos by the assay. Grouped by labels if set, otherwise `NA`. First tab shows plots using (in average) 24900 oligos with a minimum number of 10 barcodes. Second tab shows all 53691 oligos that have assigned barcodes.

- Threshold 10
- All


##### Source Code

```
---
title: "MPRAsnakeflow experiment QC report"
page-layout: full
format:
  html:
    code-fold: true
    code-tools: true
    embed-resources: true
    anchor-sections: true
    smooth-scroll: true
    toc: true
theme:
  light: flatly
  dark: darkly
jupyter: python3

---


```{python}
#| echo: false
# Imports
from IPython.display import display, Image, Markdown
import pandas as pd
import json
```

```{python}
#| tags: [parameters]
#| echo: false
```


```{python}
#| echo: false
oligo_cor_merged_table_thresh = pd.read_csv(f"{workdir}/{statistics_all_oligo_cor_thresh}", sep='\t')
oligo_cor_merged_table_all = pd.read_csv(f"{workdir}/{statistics_all_oligo_cor_all}", sep='\t')

dna_spear = oligo_cor_merged_table_thresh['DNA_spearman'].min()
rna_spear = oligo_cor_merged_table_thresh['RNA_spearman'].min()
ratio_spear = oligo_cor_merged_table_thresh['Ratio_spearman'].min()
```

```{python}
#| echo: false

threshold_dna_spear = 0.85
threshold_rna_spear = 0.95
threshold_ratio_spear = 0.75

important = dna_spear < threshold_dna_spear and rna_spear < threshold_rna_spear and ratio_spear < threshold_ratio_spear
warning = dna_spear < threshold_dna_spear or rna_spear < threshold_rna_spear or ratio_spear < threshold_ratio_spear

if important:
    display(Markdown(f'''
::: {"{.callout-important}"}

## Low correlation!

Spearman correlation across replicates is low using a barcode threshold per oligo of {thresh}! Spearman correlation of DNA is {dna_spear:.2f}, RNA is {rna_spear:.2f} and DNA/RNA ratio is {ratio_spear:.2f}. Minimum allowed values are {threshold_dna_spear:.2f}, {threshold_rna_spear:.2f}, and {threshold_ratio_spear:.2f}.

:::
'''))
elif warning:
    display(Markdown(f'''
::: {"{.callout-warning}"}

## Low correlation warning!

At least one (not all) Spearman correlations across replicates is low using a barcode threshold per oligo of {thresh}! Spearman correlation of DNA is {dna_spear:.2f}, RNA is {rna_spear:.2f} and DNA/RNA ratio is {ratio_spear:.2f}. Minimum allowed values are {threshold_dna_spear:.2f}, {threshold_rna_spear:.2f}, and {threshold_ratio_spear:.2f}.

:::
'''))
else:
    display(Markdown(f'''
::: {"{.callout-tip}"}

## Good Spearman correlation across replicates!

The data has a good Spearman correlations across replicates using a barcode threshold per oligo of {thresh}! Spearman correlation of DNA is {dna_spear:.2f}, RNA is {rna_spear:.2f} and DNA/RNA ratio is {ratio_spear:.2f}.

:::
'''))

```

# Overall quality metrics

<details>
    <summary>Table explanation</summary>

- **median rna read count:** Median of RNA read count for oligos that passed filtering to determine sufficient coverage in terms of read count. Value is the median of all replicates.
- **median barcodes passing filtering:** Median number of barcodes across tested sequences that passed filtering to determine if there was sufficient barcode to oligo coverage. Value is the median of all replicates.
- **pearson correlation:** The correlation of log2 RNA/DNA ratios across tested sequences as a measure of replicable activity signal. Value is the median of replicate comparisons using only oligos with >= `{python} thresh` barcodes.
- **fraction oligos passing:** Fraction of tested sequences that passed filtering of the mappable sequences to determine if the designed library was sufficiently recovered. Value is the median of all replicates and using only oligos with >= `{python} thresh` barcodes.

</details>

```{python}
#| echo: false
with open(f"{workdir}/{qc_metrics_file}", 'r') as file:
    qc_data = json.load(file)
df_qc = pd.DataFrame([qc_data])
df_qc.columns = df_qc.columns.str.replace("_", " ", regex=False)
#df_qc.columns = ['Value']
df_qc.style.hide(axis="index").format(precision=2)
```

# DNA over RNA counts

Plotting normalized counts of DNA vs RNA  (median across replicates). Only oligos within all replicates are shown. We should see a variation within the RNA count data (along the y axis). If count data between RNA and DNA is highly correlated (e.g. follows the identity line) there is no variation between designed oligos. This is an indication that RNA is inflated with DNA and the DNA digestion before creating cDNA did not work as expected.

::: {.panel-tabset}

## Threshold `{python} thresh`
```{python}
#| echo: false
Image(f"{workdir}/{dna_over_rna_thresh_plot}")
```

## All
```{python}
#| echo: false
Image(f"{workdir}/{dna_over_rna_plot}")
```

:::

# Oligo correlation

Oligo correlation plots of DNA, RNA and DNA/RNA ratios across replicates. First tab shows plots using (in average) `{python} "%d" % oligo_cor_merged_table_thresh['number_Oligos_Joined'].mean()` oligos with a minimum number of  `{python} thresh` barcodes. Second tab shows all `{python} "%d" % oligo_cor_merged_table_all['number_Oligos_Joined'].mean()` oligos that have assigned barcodes.

```{python}
#| echo: false

def filter_table(df):
    df = df.round(2).drop(columns=['DNA_pearson','RNA_pearson', 'Ratio_pearson', 'fraction_Oligos_ReplicateA', 'fraction_Oligos_ReplicateB', 'NormSymmetry'])
    df.columns = df.columns.str.replace("_", " ", regex=False).str.replace("Replicate", " ", regex=False).str.replace("number ", "#", regex=False)
    return(df)
```


::: {.panel-tabset}

## Threshold `{python} thresh`
```{python}
#| echo: false
oligo_cor_merged_table_thresh_filter = filter_table(oligo_cor_merged_table_thresh)
oligo_cor_merged_table_thresh_filter.style.hide(axis="index").format( precision=2) 
```

## All
```{python}
#| echo: false
oligo_cor_merged_table_all_filter = filter_table(oligo_cor_merged_table_all)
oligo_cor_merged_table_all_filter.style.hide(axis="index").format( precision=2) 
```

:::

::: {.panel-tabset}

## Threshold `{python} thresh`

::: {.panel-tabset}

### DNA
```{python}
#| echo: false
display(Image(f"{workdir}/{dna_oligo_coor_min_thre_plot }"))
```

### RNA
```{python}
#| echo: false
display(Image(f"{workdir}/{rna_oligo_coor_min_thre_plot}"))
```

### Ratio
```{python}
#| echo: false
display(Image(f"{workdir}/{ratio_oligo_min_thre_plot}"))
```

:::


## All

::: {.panel-tabset}

### DNA
```{python}
#| echo: false
display(Image(f"{workdir}/{dna_oligo_coor_plot}"))
```

### RNA
```{python}
#| echo: false
display(Image(f"{workdir}/{rna_oligo_coor_plot}"))
```

### Ratio
```{python}
#| echo: false
display(Image(f"{workdir}/{ratio_oligo_coor_plot}"))
```

:::

:::

# Experiment statistic

```{python}
#| echo: false

df_merged = pd.read_csv(f"{workdir}/{statistics_all_merged}", sep='\t').round(2)
n_oligos = df_merged['oligos design'].iloc[0]
n_barcodes = df_merged['barcodes design'].iloc[0]

avg_oligos = df_merged['oligos dna/rna'].mean()
avg_matched_barcodes =  df_merged['matched barcodes'].mean()
avg_unknown_barcodes =  df_merged['unknown barcodes dna/rna'].mean()

```


The total number of oligos in this experiment is `{python} f"{n_oligos}"` (defined by the assignment) with `{python} f"{n_barcodes}"` associated barcodes. 

In average across replicates we see `{python} f"{avg_oligos:.0f}"` from `{python} f"{avg_matched_barcodes:.0f}"` average barcodes in the count data and around `{python} f"{avg_unknown_barcodes:.0f}"` barcodes where not in the assignment.

::: {.panel-tabset}

# DNA/RNA merged
```{python}
#| echo: false
df_merged.drop(columns=['oligos design','barcodes design', 'barcodes dna/rna'], inplace=True)
df_merged.style.hide(axis="index").format( precision=2) 
```

# DNA/RNA individual
```{python}
#| echo: false
df_single = pd.read_csv(f"{workdir}/{statistics_all_single}", sep='\t').round(2)
df_single.style.hide(axis="index").format( precision=2) 
```

:::


# Histograms barcodes per oligo, counts per barcode

Histogramm of number of barcodes per oligo and the number of counts per barcode devidied by DNA and RNA. Median is red, mean is blue.


::: {.panel-tabset}

# Number of barcodes per oligo
```{python}
#| echo: false
display(Image(f"{workdir}/{counts_per_oligo}")) 
```


# Number of counts per DNA barcode
```{python}
#| echo: false
display(Image(f"{workdir}/{counts_per_bc_dna}")) 
```

# Number of counts per RNA barcode
```{python}
#| echo: false
display(Image(f"{workdir}/{counts_per_bc_rna}")) 
```

:::

# Activity

Violin and box plots of the log2 fold change of all oligos by the assay. Grouped by labels if set, otherwise `NA`. First tab shows plots using (in average) `{python} "%d" % oligo_cor_merged_table_thresh['number_Oligos_Joined'].mean()` oligos with a minimum number of  `{python} thresh` barcodes. Second tab shows all `{python} "%d" % oligo_cor_merged_table_all['number_Oligos_Joined'].mean()` oligos that have assigned barcodes.

::: {.panel-tabset}

## Threshold `{python} thresh`
```{python}
#| echo: false
display(Image(f"{workdir}/{activity_thresh}"))
```

## All
```{python}
#| echo: false
display(Image(f"{workdir}/{activity_all}"))
```

:::
```
