# Supplementary material for: Uniform processing and analysis of IGVF massively parallel reporter assay data with MPRAsnakeflow
Source: Genome Res. 2026 Sep;36(9):1940–51. doi: 10.1101/gr.281462.125 (PMC13534247; doi:10.1101/gr.281462.125)
Supplement: Supplementary file 6 [file Supplemental_Code_S3.zip › MPRAOligoDesign-0.1.2/MPRAOligoDesign.pdf]

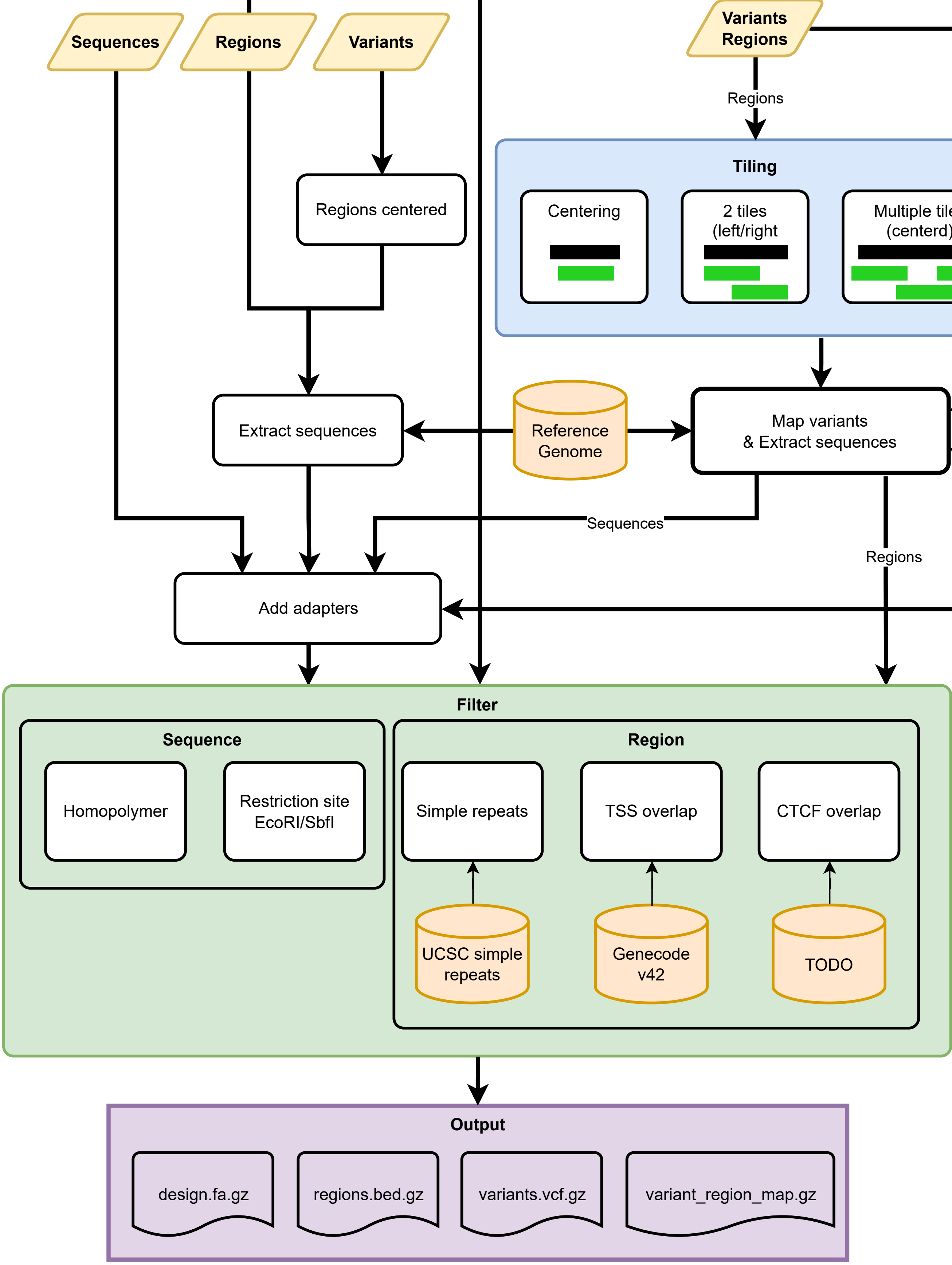

Sequences
Regions
Variants
Variants Regions
Regions
Tiling
Centering
2 tiles (left/right
Multiple tile (centerd)
Regions centered
Extract sequences
Reference Genome
Map variants & Extract sequences
Sequences
Regions
Add adapters
Filter
Sequence
Homopolymer
Restriction site EcoRI/SbfI
Region
Simple repeats
TSS overlap
CTCF overlap
UCSC simple repeats
Genecode v42
TODO
Output
design.fa.gz
regions.bed.gz
variants.vcf.gz
variant_region_map.gz

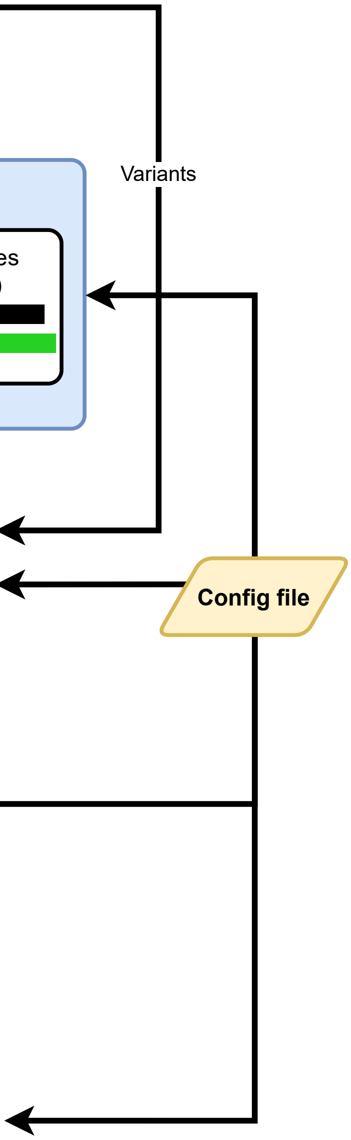
Variants
Config file
